# Children and Young People with Long COVID—Comparing Those Seen in Post-COVID Services with a Non-Hospitalised National Cohort: A Descriptive Study

**DOI:** 10.3390/children10111750

**Published:** 2023-10-28

**Authors:** Fiona Newlands, Anne-Lise Goddings, Maude Juste, Holly Boyd, Manjula D. Nugawela, Snehal M. Pinto Pereira, Emily Whelan, Elizabeth Whittaker, Terence Stephenson, Isobel Heyman, Trudie Chalder, Emma Dalrymple, CLoCk Consortium, Terry Segal, Roz Shafran

**Affiliations:** 1 Great Ormond Street Institute of Child Health, University College London, London WC1N 1EH, UK; 2 University College London Hospitals NHS Foundation Trust, London NW1 2BU, UK; 3 Division of Surgery & Interventional Science, Faculty of Medical Sciences, University College London, London WC1E 6BT, UK; 4 School of Psychology, University of Sussex, Brighton BN1 9QH, UK; 5 Department of Paediatric Infectious Diseases, Imperial College Healthcare NHS Trust, London W2 1NY, UK; 6 Institute of Psychiatry, Psychology and Neuroscience, King’s College London, London SE5 8AF, UK

**Keywords:** Post-COVID services, Long COVID, children and young people, paediatric, SARS-CoV-2

## Abstract

Background: Post-COVID services have been set up in England to treat children with ongoing symptoms of Long COVID. To date, the characteristics of children seeking treatment from these services has not been described. Purpose: (1) to describe the characteristics of children aged 11–17 referred to the Pan-London Post-COVID service and (2) to compare characteristics of these children with those taking part in the United Kingdom’s largest research study of Long COVID in children (CLoCk). Design: Data from 95 children seeking treatment from the Post-COVID service between May 2021 and August 2022 were included in the study. Their demographic characteristics, symptom burden and the impact of infection are described and compared to children from CLoCk. Results: A high proportion of children from the Post-COVID service and CLoCk reported experiencing health problems prior to the pandemic. Almost all Post-COVID service children met the research Delphi definition of Long COVID (94.6%), having multiple symptoms that impacted their lives. Symptoms were notably more severe than the participants in CLoCk. Conclusions: This study describes the characteristics of children seeking treatment for Long COVID compared to those identified in the largest longitudinal observational study to date. Post-COVID service children have more symptoms and are more severely affected by their symptoms following infection with COVID-19 than children in the CLoCk study. Research to understand predisposing factors for severity and prognostic indicators is essential to prevent this debilitating condition. Evaluation of short- and long-term outcomes of interventions by clinical services can help direct future therapy for this group.

## 1. Introduction

It is widely accepted a significant proportion of children and young people (hereafter referred to as ‘young people’) experience persistent symptoms following Severe Acute Respiratory Syndrome Coronavirus 2 (SARS-CoV-2) exposure [1]. The clinical manifestations of paediatric COVID-19 are diverse with fever and cough being amongst the most common reported symptoms [2,3]. Children who continue to experience symptoms for at least 12 weeks post infection are said to have Long COVID (also known as Post-COVID-19 Condition) [4]. Common symptoms associated with the condition are similar to acute COVID-19 and include fatigue, cognitive difficulties, headache, loss of smell [1,5]. These symptoms may fluctuate or relapse over time and have an impact on everyday functioning [4,6]. Research on Long COVID is ongoing, and several studies indicate the condition can have lasting effects on various organs and systems in the body including the kidneys, lungs, the brain and haematological characteristics [7,8,9]. Given the complexity of the condition, there is a need for specialist clinics to provide diagnosis and effective treatment options.

Specialised clinics, research initiatives and support groups have been set up across the globe to help support young people living with the condition but the availability and extent of these services vary from country to country (e.g., Ref. [10]). In June 2021, NHS England announced they were setting up 15 specialist paediatric tertiary services as part of a GBP 100 million expansion of care for those suffering from Long COVID. What is offered at each service is not uniform but the majority aim to offer multidisciplinary assessment and management with a focus on supported self-management. The announcement of services was positively received but there was a note of caution that critical evaluation was required to ensure meaningful benefit [11]. In particular, it was suggested the new services should be run as research hubs and be formally evaluated using in-practice data [11].

Although these research hubs did not come to fruition, there are now many studies exploring Long COVID in young people and systematic reviews and meta-analyses of the results have been conducted [1,12]. This research, combined with national survey data [13] yields a mixed picture, but it is clear many patients infected with SARS-CoV-2 develop long-term symptoms [14]. Given over 90% of secondary school pupils in the United Kingdom are estimated to have been exposed to SARS-CoV-2 [15], this has the potential to be extremely concerning. Even with a conservative estimate of 0.51% of 12–16-year-olds having Long COVID [16], with an estimated 4.9 million young people aged 10–16 in the United Kingdom [17], it has the potential to overwhelm services. However, these data do not detail symptom severity or impact on functionality, which may explain why prevalence estimates do not map to demand for services. We do not yet know what factors result in young people seeking treatment.

This study had two objectives: (1) describe the characteristics of young people aged 11–17 being referred to a Post-COVID service (PCS); and (2) compare these characteristics with those of young people taking part in the Children and Young People with Long COVID (CLoCk) study [18]. CLoCk is the largest matched cohort study of young people in England, in which non-hospitalised young people reported symptoms after a laboratory-confirmed SARS-CoV-2 infection and were compared to age- sex- and geographically matched controls with a laboratory-confirmed SARS-CoV-2 negative test. Demographic variables, symptoms and their impact were assessed using the questionnaire that had also been used in some of the paediatric PCSs. This paper reports on data collected from the Pan-London paediatric PCS.

Based on a combination of clinical observation and the existing literature, we had two main hypotheses. Firstly, patients referred to the PCS would have similar demographics to participants in CLoCk. Second, those referred to the PCS would experience the same range of symptoms as those in CLoCk, but have more symptoms that were more impairing, and a higher proportion of patients would meet the Delphi research definition of Long COVID [4].

## 2. Methods

### 2.1. Study Design

This is a descriptive study comparing characteristics of patients aged 11–17 referred to a PCS compared to young people in a national research study (CLoCk).

### 2.2. Setting

This paper reports on data collected from the Pan-London PCS established in April 2021. Local triage and assessment are undertaken by a paediatrician or primary care provider (if aged 16–18 yrs) to exclude other aetiological causes and secondary organ damage. This is conducted by local National Health Service (NHS) paediatricians if the young person is under 16 or by family physicians (‘General Practitioners’—GP—in England) if the patient is 16–18 years old. Where required, referrals to a PCS are made. The patient’s case is presented to the virtual multidisciplinary team at the PCS who, after discussion, recommend the patient be seen in person at the clinic or remain with their local service. Approximately 65% of patients are seen in person. The main reason for being seen at the service is severity of symptoms and impact on functioning (for example, poor educational attendance and not taking part in sports or activities).

For CLoCk, potential participants were identified using the national SARS-CoV-2 testing dataset held by the UK Health Security Agency (UKHSA) [18]. UKHSA received results of all SARS-CoV-2 PCR tests in England irrespective of the reason they were taken. Using this dataset, potential participants were approached by post and invited to take part in the study.

### 2.3. Participants

All PCS young people were asked to complete the self-report questionnaire at referral. For the PCS group, inclusion criteria were young people aged 11–17 years old who completed the questionnaire between 13 May 2021 and 17 August 2022. Patients did not require a positive SARS-CoV-2 test to be referred to the service. PCS young people who did not complete the survey were excluded from the analysis, as were PCS young people who were under 11 and over 17 years old to make the sample more comparable to CLoCk participants.

For CLoCk participants, inclusion criteria were young people aged 11–17 who had a positive PCR test between January 2021 and March 2021 and completed the questionnaire between 13 April and 3 August 2021 [5]. This comparison group were those who completed the questionnaire within 24 weeks of their PCR test to minimise the potential for recall bias. Those participants who had a negative PCR between January 2021 and March 2021 and who completed the questionnaire more than 24 weeks after their PCR test were excluded from the study.

### 2.4. Variables/Measures

The questionnaire was based on the International Severe Acute Respiratory and emerging Infection Consortium (ISARIC) working group [19] and contained demographic information including age, gender and ethnicity coded using Office of National Statistical categories [20]. It included an assessment of health prior to the pandemic, current health and health during the acute COVID-19 phase (retrospective) and standardised well-being measures. Standardised measures were selected to assess emotional wellbeing (Strengths and Difficulties Questionnaire (SDQ)) [21], quality of life and everyday functioning (EQ-5D-Y and EQ VAS) [22], fatigue (Chalder Fatigue Scale (CFS)) [23] and loneliness (UCLA-3) [24]. The Supplementary Materials and Table S1 present the questionnaire and details on how measures were interpreted.

The Index of Multiple Deprivation (IMD) was used as a proxy for socioeconomic status and was derived from the participants’ lower super output area (a small local area level-based geographic hierarchy) [25]. IMD quintiles were calculated from most (quintile 1) to least (quintile 5) deprived.

PCS patients completed the questionnaire on a paper which was then entered into Excel. A random sample of 10% of questionnaires were checked for quality assurance. CLoCk participants completed an online version of the questionnaire [18].

### 2.5. Statistical Methods

The analysis was conducted using STATA v17. Descriptive statistics were used to describe demographics (sex, age, ethnicity and region of residence), symptoms experienced before the COVID-19 pandemic, symptoms during the acute SARS-CoV-2 phase (retrospective) and at the time of completing the questionnaire (current). Histograms and Shapiro–Wilk tests were conducted to assess the distribution of data. Data were summarised as frequency and prevalence, means and standard deviations or medians and interquartile ranges (IQR) as appropriate. Two-tailed Chi-squared, Fisher’s exact or Mann–Whitney U tests were used to assess whether differences exist between PCS and CLoCk young people, with a *p*-value < 0.05 considered significant. The Benjamini–Hochberg method [26] was applied to account for the exploratory nature of analyses. *p*-values that remained significant after accounting for the false discovery rate (FDR) were reported in bold. Since the study was descriptive and explorative in nature, a power analysis was not conducted.

The completeness of the PCS questionnaire data ranged from 89% (SDQ total score) to 100% with a mean completeness ratio of 97%. The completeness of the CLoCk questionnaire data ranged from 99 to 100% with a mean completeness ratio of 100%. Where there were missing data, the reported percentage is based on the complete data for that variable.

A sub-group analysis was conducted replicating the analysis described above to compare PCS young people with CLoCk participants who met the Delphi definition of Long COVID [4].

### 2.6. Ethics

The CLoCk study was approved by the Yorkshire and The Humber—South Yorkshire Research Ethics Committee (REC reference: 21/YH/0060; IRAS project ID:293495). The project was registered as a service evaluation and was approved by the Paediatrics and Adolescent Division Quality and Safety Lead (registered on 30 March 2023).

## 3. Results

Between May 2021 and August 2022, 209 patients were referred to the PCS and 112 young people completed the questionnaire (completion rate 53.6%); 17 young people were excluded because they were under 11 or over 17 years old, leaving 95 in the final analysis. PCS young people took a test between 1 October 2020 and 1 May 2022 and completed the questionnaire between 13 May 2021 and 17 August 2022. For patients who reported a positive SARS-CoV-2 test (*n* = 70), the median time between the test and completing the questionnaire was 29.8 weeks (IQR 19.6–37.7).

Of the 23,048 PCR test-positive young people invited to take part in CLoCk, 3065 consented and completed the questionnaire within 24 weeks of their PCR test (response rate 13.3%). Young people took PCR tests that were registered on the UKHSA database between 1 January 2021 and 31 March 2021 and completed the questionnaire between 13 April 2021 and 3 August 2021 (median 14.6 weeks after PCR test).

The median age of PCS young people was 14 years (IQR 13, 15) compared to 15 years (IQR 13, 16) for CLoCk young people (Table 1). PCS consisted of more females and White young people than CLoCk (females: 67.4% (PCS), 63.5% (CLoCk); White: 84.2% (PCS), 72.8% (CLoCk); *p* ≤ 0.001 for both). Based on IMD, PCS young people were from less deprived areas than CLoCk young people, for example, 28.4% of PCS were from the ‘least deprived’ quantile compared to 20.4% from CLoCk (X2(4) = 13.4; *p* = 0.009).

A high proportion of young people reported experiencing health symptoms prior to the pandemic including allergies (PCS: 39.4%; CLoCk: 30.9%) and often feeling tired (PCS: 36.2%; CLoCk: 40.2%). There were no significant differences between groups for these symptoms (*p* > 0.05). PCS young people were significantly more likely to report experiencing problems with stomach, gut, liver, kidneys or digestion (PCS: 16.1%; CLoCk: 4.3%; *p* < 0.001), a neurological disease (PCS: 4.3%; CLoCk: 1.4%; *p* = 0.05), a physical disability (PCS: 11.7%; CLoCk: 2.2%; *p* < 0.001), a learning difficulty (PCS: 13.8%; CLoCk: 8.0%; X2(1) = 4.1; *p* < 0.04), problems with sleep (PCS: 28.3%; CLoCk: 17.9%; X2(1) = 6.4; *p* = 0.01), tummy aches (PCS: 32.3%; CLoCk: 16.3%; X2(1) = 16.4; *p* < 0.001) and other serious illness (PCS: 13.0%; CLoCk: 2.2%; *p* < 0.001). Table S2 displays the comparative statistics for symptoms prior to the pandemic.

PCS young people were more likely to report ‘some’ or ‘a lot’ of problems with daily function prior to the pandemic on the mobility (PCS: 14.0%; CLoCk: 4.4%; *p* < 0.001), self-care (PCS: 8.6%; CLoCk: 3.7%; *p* = 0.009), doing usual activities (PCS: 12.9%; CLoCk: 10.8%; *p* < 0.001) and pain (PCS: 19.4%; CLoCk: 14.7%; *p* < 0.009) domains of the EQ-5D-Y. There was no difference between the two groups on the sad/worried domain of the EQ-5D-Y (X2(2) = 2.6; *p* = 0.28).

### 3.1. Symptoms during Acute COVID-19 Phase (Retrospective)

During the acute COVID-19 phase, PCS young people reported more symptoms than those in CLoCk (median number of symptoms PCS: 10.0, IQR 7.0–14; CLoCk: 0.0, IQR 0.0–4.0; *p* < 0.001). Common symptoms are reported in Table S3.

### 3.2. Current Symptoms

A higher proportion of PCS young people met the Delphi definition of Long COVID (i.e., had at least 1 symptom which was causing functional impairment as indicated by the EQ-5D-Y) than CLoCk young people (PCS: 94.6%, 95% CI 87.8–98.2%; CLoCk: 25.6%; 95% CI 24.0–27.1%).

The majority of PCS young people (77.7%) experienced 5 or more symptoms at the time of completing the questionnaire (median 29.8 weeks after acute COVID-19 infection) compared to 13.4% of young people in CLoCK (median 14.6 weeks after PCR test-positive; X2(5) = 296.4; *p* < 0.001). The median number of symptoms reported by PCS young people was 8.0 (IQR 5.0–10.0) compared to 1.0 (0.0–3.0) in CLoCk.

The same symptoms were most common in both groups including tiredness, headaches, dizziness or light-headedness and shortness of breath; however, symptom prevalence was higher in the PCS group than in CLoCk. See Table 2.

**Figure 1:**
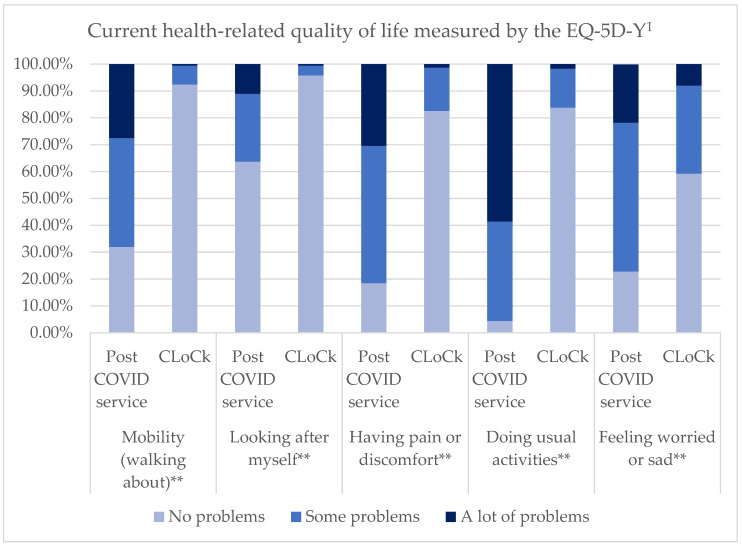
Current health-related quality of life measured by the EQ-5D-Y. ^1^ NB: The number of participants in the PCS varies due to missing data from 91 to 92. ** Significant difference between PCS and CLoCk sample at *p* < 0.001.

PCS young people were significantly more likely to report ‘some’ or ‘a lot’ of on all domains of the EQ-5D-Y (*p* < 0.001), suggesting a poorer health-related quality of life (see Figure 1 and Table S4). EQ-VAS scores were significantly lower in PCS young people indicating a poorer health-related quality of life (PCS: 35.0%, 20.0–55.0%; CLoCk: 90.0%, 80.0–95.0%; z = −14.7; *p* < 0.001).

There was no difference between the two groups in emotional well-being as assessed by total SDQ scores (median 12 (7–17) for PCS young people and 11 (6–15) for young people in CLoCk (z = −1.8; *p* = 0.07)). However, SDQ impact scores were significantly higher in PCS young people (PCS: 2 (0–5); CLoCk 0 (0-1); z = −7.7; *p* < 0.001) indicating symptoms were having a greater impairment and causing more distress.

Additionally, 96.7% of PCS young people were ‘fatigued’ compared to 35.5% of CLoCk young people (X2(1) = 136.9; *p* < 0.001). PCS young people were more likely to report feeling lonely as indicated by UCLA-3 loneliness scores (PCS: 13.2%; CLoCk: 6.5%; X2(1) = 6.4; *p* = 0.01).

### 3.3. Subgroup Analysis-PCS and CLoCk Delphi Young People

Of the 3065 test-positive respondents, 783 (25.6%) met the research Delphi definition of Long COVID [4]. As with the main CLoCk sample, Delphi young people were predominantly Female (74.2%) and White (74.3%). There were fewer CLoCk Delphi participants from the least deprived areas of England than in the main sample (16.45% and 20.4%, respectively). Table S5 presents the demographic characteristics of CLoCk Delphi young people.

The majority of CLoCk Delphi young people reported no symptoms during the acute SARS-CoV-2 phase (63.7%; median: 0; IQR: 0, 7). Symptom prevalence was highest for headaches (30.9%), tiredness (27.7%) and sore throat (27.1%).

### 3.4. Current Symptoms

**Figure 2:**
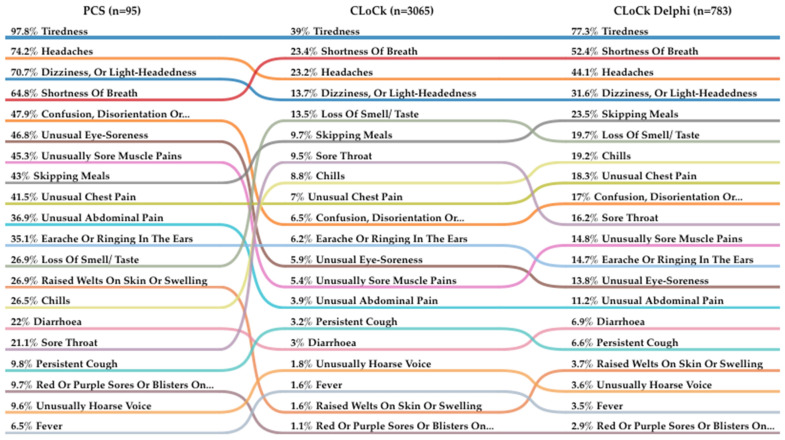
Comparison of symptom prevalence across PCS, CLoCk and CLoCk Delphi young people. Coloured lines represent the comparison of symptom prevalence across groups.

Out of the CLoCk Delphi young people, 36.0% reported experiencing 5+ symptoms compared to 77.7% of PCS young people (X2(5) = 70.9; *p* < 0.001). Common symptoms experienced by the CLoCk Delphi group were similar to those reported by PCS patients including tiredness (77.3%), shortness of breath (52.4%) and headaches (44.1%). However, symptom prevalence was lower in the CLoCk Delphi group than the PCS group. See Figure 2 for a comparison of symptom prevalence across PCS, CLoCk and CLoCk Delphi young people.

PCS young people were more likely to report problems with daily function on mobility (*p* < 0.001), self-care (*p* < 0.001), doing usual activities (X2(2) = 223.3; *p* < 0.001) and pain or discomfort (X2 (2) = 80.4; *p* < 0.001) domains of the EQ-5D-Y compared to the CLoCk Delphi group. However, there was no difference between the two groups for the sad/worried domain with 77.2% of PCS young people and 74.6% of CLoCk Delphi young people reporting ‘some problems’ or ‘a lot of problems’ (X2(2) = 3.3; *p* = 0.2).

SDQ impact scores remained significantly higher for PCS young people indicating symptoms were causing greater impairment and more distress (PCS: 2 (0–5); CLoCk Delphi: 1(0–3); (z = −2.3; *p* = 0.019)).

Out of the CLoCk Delphi young people, 70.9% were ‘fatigued’ compared to 96.6% of PCS young people (X2(1) = 26.8; *p* < 0.001). A similar proportion of both groups reported feeling lonely as captured by the UCLA-3 loneliness scale PCS: 13.2%; CLoCk Delphi: 17.4%; X2(1) = 1.0; *p* = 0.3).

## 4. Discussion

This is the first study to compare symptoms and characteristics between a population sample and a sample presenting to a PCS.

This study found a number of important differences between the PCS and CLoCk samples. Almost all PCS young people met the Delphi definition of Long COVID [4] compared to a significantly smaller proportion of CLoCk young people. Based on IMD, PCS young people were from less deprived areas than CLoCk young people and were more likely to report experiencing a range of symptoms such as problems with the stomach, gut, liver or kidneys. They were also more likely to report ‘some’ or ‘a lot’ of problems with several areas of daily function prior to the pandemic. The majority of PCS young people experienced 5 or more symptoms at the time of completing the questionnaire compared to a minority of the young people in CLoCk (13.4%). Strikingly, the median number of symptoms reported by PCS young people was 8.0 compared to 1.0 in CLoCk. Although the same symptoms were most common in both groups including tiredness, headaches, dizziness or light-headedness and shortness of breath, symptom prevalence was higher in the PCS group than in CLoCk. PCS young people were significantly more likely to have a poorer health-related quality of life with mental health symptoms having greater impact in the PCS young people than the CLoCk sample. Almost all the PCS young people were ‘fatigued’ compared to only a third of the CLoCk sample, and they were also more likely to report loneliness. Within the subsample of the CLoCk participants who met the Delphi research definition of Long COVID, symptoms were similar in nature to the PCS young people but they had far fewer of them and they were less impairing. The findings can be summarised as showing that compared to the CLoCk young people, the PCS young people had more symptoms, and those symptoms were more severe and having a greater negative impact.

The findings from this study should be viewed within the context of relevant existing literature. Systematic reviews of paediatric Long COVID and adult Long COVID typically report similar symptom profiles as to those found in the current PCS and CLoCk samples [1,27]. However, such reviews have grouped together young people recruited from different sources. Our finding that PCS young people experienced more symptoms that were having a greater impact than those in CLoCk is in line with other studies detailing the severity and long-lasting nature of symptoms experienced by patients presenting at clinics including Pulmonary Circulation Dysfunction [28] and morphologic abnormalities [29]. The finding that PCS young people reported significantly more symptoms during the acute COVID-19 phase than CLoCk young people, with the majority experiencing more than 5 symptoms at onset, aligns with studies in adult populations suggesting the presence of multiple symptoms at disease onset is predictive of Long COVID [30,31,32]. There are many possible explanations for this, including increased viral load. Although no specific biomarkers have yet been established that differentiate Long COVID from other disease entities, it is hoped that sensitive and reliable diagnostic biomarkers will emerge which may further help identify which children are in need of clinical interventions [33]. Monitoring young people reporting multiple symptoms during infection may also enable early intervention and support.

The high proportion of PCS young people reporting symptoms prior to the pandemic is congruous with research suggesting health pre-pandemic is associated with Long COVID [34,35]. Young people experiencing poor health prior to the pandemic may find it more challenging to function with the burden of additional symptoms. Additionally, experiencing poor health prior to the pandemic could be indicative of a pre-existing condition [36]. We cannot rule out the high prevalence of symptoms reported prior to the pandemic in retrospectively describing health has led to recall bias. Moreover, chronic non-specific symptoms have been experienced by young people in multiple studies prior to the pandemic and may be typical for this age group. For example, fatigue has been described in up to 40% of one cohort of young people pre-pandemic [37].

Some additional findings have important implications for clinical services, in particular, that PCS young people were from less deprived areas than CLoCk young people. This could be because the CLoCk sample was recruited nationally whereas the PCS young people were attending the Pan-London service. Should the results be replicated across different PCS services, it is important to consider methods to ensure equality of access. Self-referral to such services may be an option to consider to reduce inequalities as has been the case in other areas of health [38]. Self-referral may also be an opportunity to address the data from CLoCk suggesting there is a large proportion of young people experiencing symptoms more than 3 months after infection who are not being referred to a PCS. This could suggest the majority of young people who meet the definition do not need specialist care and are self-managing or being managed through local services. Alternatively, it could indicate an unmet need and young people who require treatment are npt receiving it. Overall, only 3.8% of young people in a study related to CLoCk but infected with the Omicron variant reported seeing a GP for their COVID-related symptoms and less than 1% had stayed overnight due to COVID-related symptoms in the six months since the original infection [39]. These findings would indicate that the former explanation i.e., the majority of infected young people are self-managing, is the more likely one, facilitated by programmes such as ‘your COVID recovery’ by the NHS [40]. However, increasing access to services for young people with Long COVID via self-referral would ensure those in need are able to be treated appropriately.

### Limitations

This study has several limitations. The two samples completed the questionnaire over different time periods, with some overlap in the time of infection. Samples were not matched in terms of demographic variables and duration between the test or contracting the virus and completing the questionnaire. This highlights challenges in comparing a research-based sample and a clinical group where the time from symptoms to presentation in a tertiary service is likely longer than 3 months. Long COVID was a new diagnosis when the questionnaire was designed, and some symptoms were not yet recognised. As a result, ‘brain fog’ is not captured as a symptom in the questionnaire. This study benchmarks a single clinic audit against data collected in a national survey and therefore cannot be generalised to other populations. Finally, PCS young people were included if they filled in the questionnaire, which was a self-selected group accounting for 53.0% and may infer bias. This also applies to the CLoCk sample which reported a response of 13.3%.

## 5. Conclusions

This study is important as it demonstrates findings from research studies such as CLoCk cannot simply be generalised to the young people meeting referral criteria to PCS; while symptom profiles are similar, the number of symptoms experienced and their impact is far higher in the clinical sample. These findings may help focus resources on those most in need. Importantly, the focus of this study was to describe the characteristics of young people from a PCS and compare them to young people in a national research study. Further studies are required to determine causal associations. Additionally, research is needed that is methodologically rigorous and that can evaluate outcomes of intervention for young people and their families who are experiencing significant distress.

## Figures and Tables

**Table 1:** Characteristics of participants in the Post-COVID service and CLoCk.

		Post-COVID Service (*n* = 95) ^1^		CLoCk ^2^ (*n* = 3065)	
		#	%	#	%
Sex ^3^	Female	64	67.4%	1945	63.5%
	Male	29	30.5%	1120	36.5%
	Prefer not to say	2	2.1%		
Age	11	6	6.3%	283	9.2%
	12	11	11.6%	285	9.3%
	13	17	17.9%	315	10.3%
	14	24	25.3%	361	11.8%
	15	18	19.0%	477	15.6%
	16	14	14.7%	622	20.3%
	17	5	5.3%	722	23.6%
	Mean age (SD)	14.0 (1.6)		14.7 (2.0)	
	Median (IQR)	14 (13, 15)		15 (13, 16)	
Ethnicity	White	80	84.2%	2231	72.8%
	Asian/Asian/British	2	2.1%	491	16.0%
	Black/African/Caribbean/British	1	1.1%	109	3.6%
	Mixed	10	10.5%	147	4.8%
	Other	1	1.1%	60	2.0%
	Prefer not to say/unknown	1	1.1%	27	0.9%
IMD ^4^	1 (most deprived)	5	5.8%	643	21.0%
	2	18	20.7%	633	20.7%
	3	19	21.8%	571	18.6%
	4	20	23.0%	593	19.3%
	5 (least deprived)	25	28.4%	625	20.4%

^1^ NB: # varies due to missing data from 87 (for IMD) to 95 (for age, ethnicity and sex). ^2^ Children and Young People with Long COVID (CLoCk) study. ^3^ Data were provided by UKHSA who have a record of assigned sex at birth. ^4^ Index of Multiple Deprivation.

**Table 2:** Heat map demonstrating current symptom prevalence in PCS and CLoCk populations ^1^.

Symptom	PCS (n = 95) ^2^	CLoCk (n = 3065)	Statistical Test ^3^
Tiredness	97.8%	39.0%	X^2^ (1)= 127.9; ***p* < 0.001**
Headaches	74.2%	23.2%	X^2^ (1)= 126.5; ***p* < 0.001**
Dizziness or light-headedness	70.7%	13.7%	X^2^ (1)= 223.4; ***p* < 0.001**
Shortness of breath	64.8%	23.4%	X^2^ (1)= 79.1; ***p* < 0.001**
Confusion, disorientation or downiness	47.9%	6.5%	X^2^ (1)= 220.3; ***p* < 0.001**
Unusual eye soreness	46.8%	5.9%	X^2^ (1)= 229.3; ***p* < 0.001**
Unusually sore muscle pains	45.3%	5.4%	X^2^ (1)= 238.3; ***p* < 0.001**
Skipping meals	43.0%	9.7%	X^2^ (1)= 105.6; ***p* < 0.001**
Unusual chest pain	41.5%	7.0%	X^2^ (1)= 145.8; ***p* < 0.001**
Unusual abdominal pain ^4^	36.9%	3.9%	***p* < 0.001**
Earache or ringing in the ears	35.1%	6.2%	X^2^ (1)= 115.4; ***p* < 0.001**
Loss of smell/taste	26.9%	13.5%	X^2^ (1)= 13.5; ***p* < 0.001**
Raised welts on skin or swelling ^4^	26.9%	1.6%	***p* < 0.001**
Chills	26.4%	8.8%	X^2^ (1) = 32.5; ***p* < 0.001**
Diarrhoea ^4^	22.0%	3.0%	***p* < 0.001**
Sore throat	21.1%	9.5%	X^2^ (1)= 13.9; ***p* < 0.001**
Persistent cough ^4^	9.8%	3.2%	***p* = 0.003**
Unusually hoarse voice ^4^	9.6%	1.8%	***p* < 0.001**
Red or purple sores or blisters on feet ^4^	9.7%	1.1%	***p* < 0.001**
Fever ^4^	6.5%	1.6%	***p* = 0.005**

^1^ Darker colour cells represent symptoms with the highest prevalence. ^2^ NB: # varies due to missing data from 88 (shortness of breath) to 95 (sore throat). ^3^ Number of comparisons = 68; false discovery rate (FDR) = 0.0375; *p*-values presented in bold were still significant after accounting for the FDR. ^4^ Fisher’s exact test was used where assumptions for Chi-squared were not met.

## Data Availability

Data are not publicly available. All requests for CLoCk data will be reviewed by the study team to verify whether the request is subject to any intellectual property or confidentiality obligations. Requests for access to the data from this study can be submitted via email to Clock@phe.gov.uk with detailed proposals for approval. A signed data access agreement with the CLoCk team is required before accessing shared data.

## Supplementary Materials

The following supporting information can be downloaded at: https://www.mdpi.com/article/10.3390/children10111750/s1, Table S1: Information on measures included in the CLoCk questionnaire and details on how they have been dichotomised; Table S2: Symptom profile before COVID-19 pandemic in March 2020 (retrospective reports); Table S3: Symptoms during acute COVID phase; Table S4: Current health related quality of life; Table S5: Demographics of CLoCk Delphi CYP.
