# Supplementary material for: Children and Young People with Long COVID—Comparing Those Seen in Post-COVID Services with a Non-Hospitalised National Cohort: A Descriptive Study
Source: Children (Basel). 2023 Oct 28;10(11):1750. doi: 10.3390/children10111750 (PMC10670307; doi:10.3390/children10111750)
Supplement: Supplementary file 1 [file children-10-01750-s001.zip › children-2626659-supplementary.pdf]

Supplementary materials

Table of Contents

Questionnaire 2

Table S1. Information on measures included in the CLoCk questionnaire and details on how they have been dichotomised 26

Table S2: Symptom profile before COVID-19 pandemic in March 2020 (retrospective reports) 27

Table S3: Symptoms during acute COVID phase 29

Table S4: Current health related quality of life 31

Table S5: Demographics of CLoCk Delphi CYP 32

References 33

Questionnaire

Health questionnaire for children and young people aged 11-18 to answer directly.

These questions are to be answered by the Young Person who had the Covid-19 test.

If you need any help, please ask a parent, relative, carer or friend to help you.

For questions that ask for a particular date, don't worry if you can't remember it exactly, just enter the closest date.

The questions **do not** need to be completed in one go but can be paused and continued at a later time.

All of the information which you provide will be kept confidential and will not be shared with anyone outside the research team studying Long Covid in young people.

Please enter the unique personal number on the front of the letter which we sent you asking you to take part:

Consent

Before starting the survey, please read the relevant consent and information sheets

Which age group does the participant belong to

- q 11-15
- q 16-18

Do you consent to take part in the study

- q Yes
- q No

About you

Your email address so that we can contact you again (this will not be shared with anyone else): ____

_

Please re-enter your email address:

____

About you

Please tick this box to confirm the email address is correct

q Yes, this email is correct

***Sex at birth:** □ Male □ Female □ Prefer not to say

***How old are you? (in years):**

How tall are you? _______(□ cm □ metres □ feet/inches) □ Not sure

What is your weight now? ___________(□ kg □ stone □ lbs) □ Not sure

***What did you weigh before your Covid-19 test?** ________ (□ kg □ stone □ lbs) □ Not sure

***What is your postcode? ___________**

***How many brothers and sisters do you have?**

*Ethnicity: **What is your ethnic group?**

Choose one option that best describes your ethnicity:

White

1. English/Welsh/Scottish/Northern Irish/British
2. Irish
3. Gypsy or Irish Traveller
4. Any other White background

Mixed/Multiple ethnic groups

5. White and Black Caribbean
6. White and Black African
7. White and Asian
8. Any other Mixed/Multiple ethnic background

Asian/Asian British

9. Indian
10. Pakistani
11. Bangladeshi
12. Chinese
13. Any other Asian background

Black/African/Caribbean/Black British

14. African
15. Caribbean
16. Any other Black/African/Caribbean background

Other ethnic group

17. Arab
18. Any other ethnic group
19. Prefer not to say

If other, please describe

____

Just before the Covid-19 pandemic in early March 2020 were you experiencing:-

Asthma?	Yes/No
Lung disease other than asthma?	Yes/No<br>If yes, please describe
Allergy problems (skin eczema, hay fever, food allergies)	Yes/No<br>If yes, please describe
Problems with your stomach, gut, liver, kidneys or digestion?	Yes/No<br>If yes, please describe
A neurological disease*(one that affects the brain or nervous system e.g. epilepsy)	Yes/No<br>If yes, please describe
Any physical disability	Yes/No<br>If yes, please describe
Learning difficulties at school	Yes/No<br>If yes, please describe
Did you have an Educational Care and Health Plan (ECHP) giving extra support at school?	Yes/No
Problems with your sleep, including getting to sleep, waking in the night or waking early?	Yes/No<br>If yes, please describe
Problems with your eating including eating too much, eating too little or eating in an uncontrolled way? (Binge eating)	Yes/No<br>If yes, please describe
A loss of interest or pleasure in doing things?	Yes/No
If yes, how often	Half the time, More than half the time, Nearly always
Feeling down, depressed or hopeless	Yes/No
If yes, how often	Half the time, More than half the time, Nearly always
Worrying a lot about bad things or the future	Yes/No
If yes, how often	Sometimes, Often, Always
Problems with headaches	Yes/No
If yes, how often	Sometimes, Often, Always
Problems with tummy aches	Yes/No
If yes, how often	Sometimes, Often, Always

Problems with friendships	Yes/No
Do you often feel very tired?	Yes/No
If yes, how often	Half the time, More than half the time, Nearly always
Any other serious ill health?	Yes/No<br>If yes, please describe
**Just before the Covid-19 pandemic in early March 2020 were you:-**	
Smoking?	Yes/No<br>How many per day on average?
Using e-cigarettes?	Yes/No<br>How many uses per day on average?

How was your <u>physical health</u> in general before your Covid-19 test?

□ Very poor □ Poor □ Ok □ Good □ Very good

If you ticked poor or very poor, please tell us why:

How was your <u>mental health</u> in general before your Covid-19 test?

□ Very poor □ Poor □ Ok □ Good □ Very good

If you ticked poor or very poor, please tell us why:

Before your Covid-19 test, were you taking any medicine given by your doctor (e.g., to help manage your concentration?)

Yes/No

Please list the medicines you were taking? (you can ask an adult for help)

Before your Covid-19 test, were you getting any help such as ‘talking therapy’ for your mental health? E.g. talking to the school counsellor

Yes/No

What kind of help?

About your Covid-19 test

Have you had a positive COVID-19 test result?

□ Yes □ No

How many positive COVID-19 test results have you had?

What was the date of your first positive COVID-19 test?

If more than 1: What was the date of your most recent positive COVID-19 test?

If your tests have been negative, do you believe that you had COVID-19? (please answer these in relation to your last Covid-19 test)

□ Yes □ No □ Not sure □ Not applicable

What was the reason for your most recent Covid-19 test?

I had some symptoms.

I was near someone who had tested positive

School testing

Other

In the last four weeks, how many school days (online or in person) in total did you miss because of symptoms of COVID-19

□ None □ 1-2 days □ 3-5 days □ 6-10 days □ 11-15 days □ More than 15 days

If you had symptoms, please answer the following questions

When did you first notice them?

How long did they last?

□ A day or less □ a few days □ about a week □ more than a week □ A couple of weeks or more

How bad were the symptoms at their worst?

□ Not very – I could carry on doing things □ a little – I felt a little bit poorly □ quite bad – I had to go to bed sometimes □ very bad – I couldn't do much □ Extremely bad – I couldn't do anything

What symptoms did you have? Check all that apply. [Items from section 4]

q Fever

- q chills or shivers (feeling too cold)
- q persistent cough (coughing a lot for more than an hour, or 3 or more coughing episodes in 24 hours)
- q unusual fatigue/tiredness
- q unusual shortness of breath
- q loss of smell/taste
- q unusually hoarse voice
- q unusual chest pain or tightness in your chest
- q unusual abdominal pain
- q diarrhoea
- q headache
- q confusion, disorientation or drowsiness
- q unusual eye-soreness or discomfort (e.g. light sensitivity, excessive tears, or pink/red eye)
- q skipping meals
- q dizziness or light-headedness
- q sore throat
- q unusual strong muscle pains
- q earache or ringing in your ears (tinnitus)
- q raised, red, itchy welts on the skin or sudden swelling of the face or lips
- q red/purple sores or blisters on your feet, including your toes
- q other

If other, please state

____

What were your *main* symptoms?

- q Fever
- q chills or shivers (feeling too cold)
- q persistent cough (coughing a lot for more than an hour, or 3 or more coughing episodes in 24 hours)

- q unusual fatigue/tiredness
- q unusual shortness of breath
- q loss of smell/taste
- q unusually hoarse voice
- q unusual chest pain or tightness in your chest
- q unusual abdominal pain
- q diarrhoea
- q headache
- q confusion, disorientation or drowsiness
- q unusual eye-soreness or discomfort (e.g. light sensitivity, excessive tears, or pink/red eye)
- q skipping meals
- q dizziness or light-headedness
- q sore throat
- q unusual strong muscle pains
- q earache or ringing in your ears (tinnitus)
- q raised, red, itchy welts on the skin or sudden swelling of the face or lips
- q red/purple sores or blisters on your feet, including your toes
- q other

If other, please state

____

____

Did you/your parent talk to the doctor about your Covid-19 symptoms? □ Yes □ No

Did you go to hospital about your Covid-19? □ Yes □ No

Did you have to stay overnight in hospital for Covid-19? □ Yes □ No

Have you had a vaccination against COVID-19?

□ Yes □ No

About your health at the moment

If you have had symptoms of COVID-19, how much do you agree with the following statement?

"I have fully recovered from COVID-19"

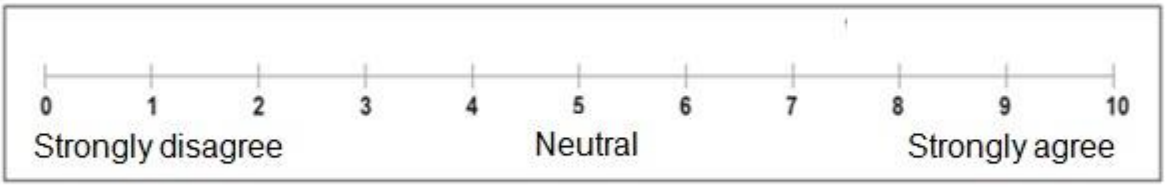

How do you feel right now?	I feel as healthy as normal<br><br>I am not feeling quite right
Do you have a fever?	Yes/No
Do you feel chills or shivers (feel too cold)?	Yes/No
If you are able to measure it, what is your temperature?	
Do you have a persistent cough (coughing a lot for more than an hour, or 3 or more coughing episodes in 24 hours)?	Yes/No
Are you experiencing unusual fatigue/tiredness?	No<br><br>Mild fatigue<br><br>Severe fatigue - I struggle to get out of bed
Are you experiencing problems with your sleep, including getting to sleep, waking in the night or waking early?	Yes/No
If yes, please describe	
Are you experiencing unusual shortness of breath?	No<br><br>Yes, **mild** symptoms - slight shortness of breath during ordinary activity<br><br>Yes, **significant** symptoms - breathing is comfortable only at rest<br><br>Yes, **severe** symptoms - breathing is difficult even at rest

What are your current symptoms? (Please tick all that apply)

loss of smell/taste

unusually hoarse voice

unusual chest pain or tightness in your chest

unusual abdominal pain

diarrhoea

headache

confusion, disorientation or drowsiness

unusual eye-soreness or discomfort (e.g. light sensitivity, excessive tears, or pink/red eye)

skipping meals

dizziness or light-headedness

sore throat

unusual strong muscle pains

earache or ringing in your ears (tinnitus)

raised, red, itchy welts on the skin or sudden swelling of the face or lips

red/purple sores or blisters on your feet, including your toes

no symptoms

other

Are there other important symptoms you want to share with us?

Since the start of your COVID-19 symptoms, have you had a period longer than one week with none of the above symptoms at all (where you were back to how you were pre-COVID)	Yes (I have had a period of one week or more since my test with none of the above symptoms)<br><br>No (My symptoms have been continuous since Covid test)<br><br>Not applicable

How you feel about your overall health

Describing your health ***BEFORE your COVID-19 test***

Under each heading, please tick the ONE box that describes **your health BEFORE your COVID-19 test**

Mobility (walking about)

I had no problems walking about	
I had some problems walking about	
I had a lot of problems walking about	

Looking after myself

I had no problems washing or dressing myself	
I had some problems washing or dressing myself	
I had a lot of problems washing or dressing myself	

Doing usual activities (for example, going to school, hobbies, sports, playing, doing things with family or friends)

I had no problems doing my usual activities	
I had some problems doing my usual activities	
I had a lot of problems doing my usual activities	

Having pain or discomfort

I had no pain or discomfort	
I had some pain or discomfort	
I had a lot of pain or discomfort	

Feeling worried, sad or unhappy

I was not worried, sad or unhappy	
I was a bit worried, sad or unhappy	
I was very worried, sad or unhappy	

*Describing your health **<u>TODAY</u>***

Under each heading, please tick the ONE box that describes **<u>your health TODAY</u>**

Mobility (walking about)

I have <u>no</u> problems walking about	
I have <u>some</u> problems walking about	
I have <u>a lot</u> of problems walking about	

Looking after myself

I have <u>no</u> problems washing or dressing myself	
I have <u>some</u> problems washing or dressing myself	
I have <u>a lot of</u> problems washing or dressing myself	

Doing usual activities (for example, going to school, hobbies, sports, playing, doing things with family or friends)

I have no problems doing my usual activities	
I have some problems doing my usual activities	
I have a lot of problems doing my usual activities	

Having pain or discomfort

I have no pain or discomfort	
I have some pain or discomfort	
I have a lot of pain or discomfort	

Feeling worried, sad or unhappy

I am not worried, sad or unhappy	
I am a bit worried, sad or unhappy	
I am very worried, sad or unhappy	

BEFORE your COVID-19 test

Questions	Hardly Ever or Never	Some of the time	Often
1. How often did you feel that you have no one to talk to?	1	2	3
2. How often did you feel left out?	1	2	3
3. How often did you feel alone?	1	2	3

	Often/Always	Some of the time	Occasionally	Hardly Ever	Never
4. How often did you feel lonely?	1	2	3	4	5

TODAY

Questions	Hardly Ever or Never	Some of the time	Often
1. How often do you feel that you have no one to talk to?	1	2	3
2. How often do you feel left out?	1	2	3

3. How often do you feel alone?	1	2	3

	Often/Always	Some of the time	Occasionally	Hardly Ever	Never
4. How often do you feel lonely?	1	2	3	4	5

We would like to know how good or bad your health was **BEFORE your Covid-19 test*** and how it is **TODAY**

This scale is numbered from 0 to 100%

100% means the best health you can think of

0% means the worst health you can think of.

Please look at the scale and draw a circle to **select the number** for your health **BEFORE your Covid-19** test and your health **TODAY**

Before Covid-19 Test

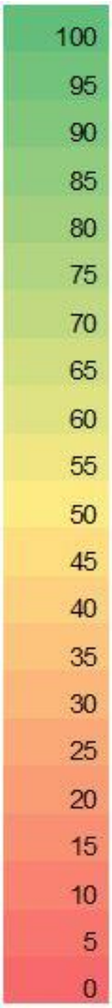

Today

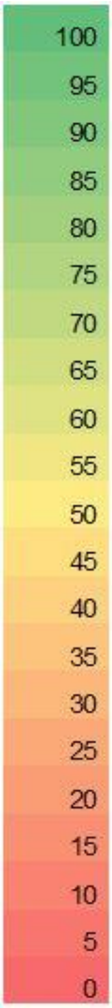

Covid-19 and your family

Has Covid-19 affected your family members and if so, can you tell us who?

	**In your house**				**In your extended family (Grandparents, aunts, uncles etc)**			
	Yes	*No*	*Don't know*	*Who?*	*Yes*	*No*	*Don't know*	*Who?*
Has anyone tested positive for Covid-19?								
Has anyone been to hospital with Covid-19?								
Has anyone been in intensive care (ICU) with Covid-19?								
Has anyone died from Covid-19?								
Does anyone have ongoing problems from Covid-19?								

Wellbeing

Strengths and Difficulties Questionnaire

For the next set of questions, please mark the box for Not True, Somewhat True or Certainly True. It would help us if you answered all items as best you can even if you are not absolutely certain or the question seems daft! Please give your answers on the basis of how things have been for you over the **PAST MONTH**.

	Not True	Somewhat True	Certainly True
I try to be nice to other people. I care about their feelings			
I am restless, I cannot stay still for long			

I get a lot of headaches, stomach-aches, or sickness			
I usually share with others (food, games, pens etc.)			
I get very angry and often lose my temper			
I am usually on my own. I generally play alone or keep to myself			
I usually do as I am told			
I worry a lot			
I am helpful if someone is hurt, upset, or feeling ill			
I am constantly fidgeting or squirming			
I have one good friend or more			
I fight a lot. I can make other people do what I want			
I am often unhappy, down-hearted or tearful			
Other people my age generally like me			

I am easily distracted, I find it difficult to concentrate			
I am nervous in new situations. I easily lose confidence			
I am kind to younger children			
I am often accused of lying or cheating			
Other children or young people pick on me or bully me			
I often volunteer to help others (parents, teachers, children)			
I think before I do things			
I take things that are not mine from home, school or elsewhere			
I get on better with adults than with people my own age			
I have many fears, I am easily scared			
I finish the work I'm doing. My attention is good			

Overall, do you think that you have difficulties in one or more of the following areas: emotions, concentration, behaviour or being able to get on with other people?

No	Yes – minor difficulties	Yes – definite difficulties	Yes – severe difficulties

If you have answered “Yes”, please answer the following questions about these difficulties:

- How long have these difficulties been present?

Less than a month	1-5 months	6-12 months	Over a year

- Do the difficulties upset or distress you?

Not at all	Only a little	Quite a lot	A great deal

- Do the difficulties interfere with your everyday life in the following areas?

	Not at all	Only a little	Quite a lot	A great deal
HOME LIFE				
FRIENDSHIPS				
CLASSROOM LEARNING				
LEISURE ACTIVITIES				

- Do the difficulties make it harder for those around you (family, friends, teachers, etc.)?

Not at all	Only a little	Quite a lot	A great deal

Chalder Fatigue Scale

We would like to know more about any problems you have had with feeling tired, weak or lacking in energy in the **LAST MONTH**. Please answer ALL the questions by ticking the answer which applies to you most closely. If you have been feeling tired for a long while, then compare yourself to how you felt when you were last well.

	less than usual	***no more than usual***	***more than usual***	***much more than usual***
do you have problems with tiredness?				
do you need to rest more?				
do you feel sleepy or drowsy?				
do you have problems starting things?				
do you lack energy?				
do you have less strength in your muscles?				
do you feel weak?				
do you have difficulties concentrating?				
do you make slips of the tongue when speaking?				
do you find it more difficult to find the right word?				
	better than usual	***no worse than usual***	***worse than usual***	***much worse than usual***
how is your memory?				

Please tick the box that best describes your experience of each over the last 2 weeks.

STATEMENTS	None of the time	Rarely	Some of the time	Often	All of the time
I've been feeling optimistic about the future	1	2	3	4	5
I've been feeling useful	1	2	3	4	5
I've been feeling relaxed	1	2	3	4	5
I've been dealing with problems well	1	2	3	4	5
I've been thinking clearly	1	2	3	4	5
I've been feeling close to other people	1	2	3	4	5
I've been able to make up my own mind about things	1	2	3	4	5

FINAL QUESTION

Please use this space if there is there anything else you would like to tell us about your health or how the pandemic or lockdown have affected you.

This research study cannot offer treatment. If you feel you would like some help, please contact

- your GP
- ChildLine www.childline.org.uk
- NHS 111 111.nhs.uk/, or call on 111
- Shout giveusashout.org/, or text 85258

Thank you

Thank you so much for completing this questionnaire.

We will send you the same questionnaire but with fewer questions in a few weeks.

You will be asked to complete the questionnaire two or three more times.

At the end of the study (in about 2 years) after completing all of the questionnaires, you will receive a £25 voucher.

Please indicate which voucher you would prefer

Amazon

LOVE2SHOP

Table S1. Information on measures included in the CLoCk questionnaire and details on how they have been dichotomised

Measure	Details (including how measures have been dichotomised)
**21 symptoms**	Mostly assessed as present/absent
**Strengths and Difficulties Questionnaire**	25 items that are combined to form five subscales (five items each): emotional symptoms, conduct problems, hyperactivity, peer relationships and the prosocial skills subscale. Each of the 25 items are scored from 0 to 2, giving a score for each subscale ranging from 0 to 10. All the subscales, except the prosocial subscale, are summed to produce a total difficulties score ranging from 0 to 40.<br><br>A further impact subscale score indicates the impact of difficulties on CYP in terms of distress and social impairment; it is scored 0-10 with increasing impact producing a higher score.<br><br>We used established cut-off points: ≥18 (total difficulties); ≥6 (emotional symptoms), ≥5 (conduct problems), ≥7 (hyperactivity); ≥4 (peer difficulties); ≤5 (prosocial skills) and ≥2 for impact.[1]
**Quality of life/functioning (EQ-5D-Y)**	For mobility, looking after self, doing usual activities, and having pain or discomfort: experiencing some or a lot of problems; for worried/sad/unhappy: feeling very worried, sad or unhappy.[2]
**Loneliness (UCLA Loneliness Scale)**	3 items from the UCLA Loneliness Scale were summed to create a total score (range: 3-9); lonely defined as a total score ≥8.[3,4]
**Fatigue (Chalder Fatigue Scale; CFQ-11)**	11 items were each dichotomised (4-point scale and coded as: 0,0,1,1). The 11 dichotomised variables were summed to a 0-11 scale. Fatigue was defined using the established cut-off of ≥ 4.[5,6]
**Long COVID**	Using data from the questionnaire on the 21 symptoms and the EQ-5D-Y scale (see details above), the Delphi research definition of Long COVID was operationalized as having at least 1 symptom and experiencing some/a lot of problems with respect to mobility, self-care, doing usual activities or having pain/discomfort or feeling very worried/sad.

Table S2: Symptom profile before COVID-19 pandemic in March 2020 (retrospective reports)

	PCS (n=95)[1]	CLoCk (n=3,065)	Statistical test[2]
Symptoms prior to COVID-19 pandemic			
Allergy problems	39.4%	30.9%	X2 (1)=3.1; p=0.08
Often feeling tired	36.2%	40.2%	X2 (1)=0.6; p=0.4
Worrying a lot about bad things or the future	34.0%	37.2%	X2 (1)=0.4; p=0.5
Problems with headaches	32.3%	26.3%	X2 (1)=1.6; p= 0.2
Problems with tummy aches	32.3%	16.3%	X2 (1)=16.4; **p<0.001**
Problems with sleep	28.3%	17.9%	X2 (1)=6.4; **p=0.01**
Feeling down, depressed or hopeless	21.5%	24.9%	X2 (1)=0.6; p=0.5
Problems with eating	16.1%	12.9%	X2 (1)=0.8; p=0.3
Problems with stomach, gut, liver, kidneys or digestion	16.1%	4.3%	two-tailed **p<0.001**
A loss of interest or pleasure	16.1%	21.0%	X2 (1)=1.3; p=0.3
Learning difficulty	13.8%	8.0%	X2 (1)=4.1; p=0.04
Other serious illness	13.0%	2.2%	two-tailed **p<0.001**
Physical disability	11.7%	2.2%	two-tailed **p<0.001**
Problems with friendships	11.7%	17.0%	X2 (1)=1.8; p=0.2
Asthma	9.8%	10.5%	X2 (1)=0.05; p=0.8
Educational Care Health Plan	8.5%	5.4%	X2 (1)=1.7; p=0.2
Neurological disease	4.3%	1.4%	two-tailed **p=0.05**
Lung disease	1.1%	0.3%	two-tailed p=0.3

1. NB: # varies due to missing data from 92 to 94
2. Number of comparisons= 68; False discovery rate (FDR)= 0.0375; p-values presented in bold were still significant after accounting for the FDR.

Table S3: Symptoms during acute COVID phase

	Post COVID service (n=95)[1]		CLoCk- all positive (n=3,065)		Sub-group CLoCk- long COVID (n=783)	
	#	%	#	%	#	%
No reported symptom	3	3.3%	1981	64.6%	499	63.7%
1 symptom	2	2.2%	60	2.0%	6	0.8%
2 symptoms	2	2.2%	88	2.9%	5	0.6%
3 symptoms	2	2.2%	101	3.3%	7	0.9%
4 symptoms	3	3.3%	109	3.6%	23	2.9%
5+ symptoms	80	87.0%	726	23.7%	243	31.0%
Mean (SD)	9.9 (4.5)		2.4 (3.9)		3.3 (5.0)	
Median	10 (7, 14)		0 (0, 4)		0 (0, 7)	
Fever	59	64.1%	548	17.9%	174	22.2%
Chills	57	62.0%	461	15.0%	153	19.5%
Persistent cough	39	42.4%	476	15.5%	159	20.3%
Tiredness	79	85.9%	696	22.7%	217	27.7%
Shortness of breath	45	48.9%	354	11.5%	153	19.5%
Loss of smell/ taste	48	52.2%	631	20.6%	182	23.2%
Unusually hoarse voice	19	20.7%	145	4.7%	66	8.4%
Unusual chest pain	39	42.4%	280	9.1%	135	17.2%
Unusual abdominal pain	40	43.5%	138	4.5%	67	8.6%
Diarrhoea	27	29.0%	166	5.4%	68	8.7%
Headaches	74	80.4%	806	26.3%	242	30.9%
Confusion, disorientation or downiness	47	51.1%	225	7.3%	115	14.69%
Unusual eye-soreness	38	41.3%	185	6.0%	77	9.8%
Skipping meals	48	52.2%	360	11.7%	147	18.8%
Dizziness, or light-	59	64.8%	462	15.1%	178	22.7%

headedness						
Sore throat	57	62.0%	687	22.4%	212	27.1%
Unusually sore muscle pains	44	47.8%	338	11.0%	136	17.4%
Earache or ringing in the ears	32	34.8%	155	5.1%	77	9.8%
Raised welts on skin or swelling	22	24.4%	35	1.1%	20	2.6%
Red or purple sores or blisters on feet	7	7.8%	21	0.7%	12	1.5%
Other	29	32.2%	73	2.4%	25	3.2%

1. NB: # varies due to missing data from 90 to 93

Table S4: Current health related quality of life

EQ 5D Y: mobility	Post COVID service (n=95)[1]	CLoCk- all positive (n=3,065)	Comparative statistics[2]
No/ none	31.9%	92.4%	**$p<0.001$**
Some problems	40.6%	7.1%	
A lot of problems	27.5%	0.5%	
EQ 5D Y: self-care*			
No/ none	63.7%	95.8%	**$p<0.001$**
Some problems	25.3%	3.7%	
A lot of problems	11.00%	0.5%	
EQ 5D Y: doing usual activities			
No/ none	4.4%	83.8%	**$p<0.001$**
Some problems	36.9%	14.5%	
A lot of problems	58.7%	1.7%	
EQ 5D Y: pain			
No/ none	18.5%	82.6%	**$p<0.001$**
Some problems	51.1%	16.1%	
A lot of problems	30.4%	1.3%	
EQ 5D Y: sad/worried			
No/ none	22.8%	59.2%	X^2 (2)=53.9; **$p<0.001$**
Some problems	55.4%	32.8%	
A lot of problems	21.7%	8.0%	

1. NB: # varies due to missing data from 91 to 92
2. Number of comparisons= 68; False discovery rate (FDR)= 0.0375; p-values presented in bold were still significant after accounting for the FDR.

Table S5: Demographics of CLoCk Delphi CYP

		Post COVID service (n=95)		CLoCk- all positive (n=3,065)		Sub-group CLoCk- long COVID (n=783)	
		#	%	#	%	#	%
Sex	Female	64	67.4%	1,945	63.5%	584	74.6%
	Male	29	30.5%	1,120	36.5%	199	25.4%
	Prefer not to say	2	2.1%	-	-	-	-
Age	11	6	6.3%	283	9.2%	53	6.8%
	12	11	11.6%	285	9.3%	49	6.3%
	13	17	17.9%	315	10.3%	68	8.7%
	14	24	25.3%	361	11.8%	87	11.1%
	15	18	19.0%	477	15.6%	126	16.1%
	16	14	14.7%	622	20.3%	185	23.6%
	17	5	5.3%	722	23.6%	215	27.5%
	Mean age (SD)	14.0 (1.6)		14.7 (1.9)		15 (1.8)	
	Median (IQR)	14 (13, 15)		15 (13, 16)		16 (14, 17)	
Ethnicity	White	80	84.2%	2,231	72.8%	582	74.3%
	Asian/ Asian/ British	2	2.1%	491	16.0%	113	14.4%
	Black/African/Caribbean/British	1	1.1%	109	3.6%	26	3.3%
	Mixed	10	10.5%	147	4.8%	45	5.7%
	Other	1	1.1%	60	2.0%	9	1.1%
	Prefer not to say/unknown	1	1.1%	27	0.9%	8	1.0%

References

1 youthinmind Information for researchers and professionals about the Strengths & Difficulties Questionnaires. Available at: https://www.sdqinfo.org/norms/UKSchoolNorm.html (accessed 22 Aug 2023).

2 Wille N, Badia X, Bonsel G, *et al.* Development of the EQ-5D-Y: a child-friendly version of the EQ-5D. *Qual Life Res* 2010;**19**:875–86. doi:10.1007/s11136-010-9648-y

3 Klein EM, Zenger M, Tibubos AN, *et al.* Loneliness and its relation to mental health in the general population: Validation and norm values of a brief measure. *J Affect Disord Rep* 2021;**4**:100120. doi:10.1016/j.jadr.2021.100120

4 Office for National Statistics. Recommended national indicators of loneliness - Office for National Statistics. Off. Natl. Stat. 2018.https://www.ons.gov.uk/peoplepopulationandcommunity/wellbeing/compendium/nationalmeasurementofloneliness/2018/recommendednationalindicatorsofloneliness (accessed 3 Mar 2023).

5 Chalder T, Berelowitz G, Pawlikowska T, *et al.* Development of a fatigue scale. *J Psychosom Res* 1993;**37**:147–53. doi:10.1016/0022-3999(93)90081-P

6 Loge JH, Ekeberg O, Kaasa S. Fatigue in the general Norwegian population: normative data and associations. *J Psychosom Res* 1998;**45**:53–65. doi:10.1016/s0022-3999(97)00291-2
